# Analyzing the determinants to accept a virtual assistant and use cases among cancer patients: a mixed methods study

**DOI:** 10.1186/s12913-022-08189-7

**Published:** 2022-07-09

**Authors:** Martien J. P. van Bussel, Gaby J. Odekerken–Schröder, Carol Ou, Rachelle R. Swart, Maria J. G. Jacobs

**Affiliations:** 1 Department of Radiation Oncology (Maastro), GROW School for Oncology, Maastricht University Medical Centre+, Maastricht, The Netherlands; 2 Department of Marketing and Supply Chain Management, Maastricht University, Maastricht, The Netherlands; 3 Tilburg School of Economics and Management, Department of Management, Tilburg University, Tilburg, The Netherlands

**Keywords:** Virtual assistants (VAs), Chatbots, Conversational agents, Healthcare, Cancer, Patients

## Abstract

**Background:**

Technological progress in artificial intelligence has led to the increasing popularity of virtual assistants, i.e., embodied or disembodied conversational agents that allow chatting with a technical system in a natural language. However, only little comprehensive research is conducted about patients' perceptions and possible applications of virtual assistant in healthcare with cancer patients. This research aims to investigate the key acceptance factors and value-adding use cases of a virtual assistant for patients diagnosed with cancer.

**Methods:**

Qualitative interviews with eight former patients and four doctors of a Dutch radiotherapy institute were conducted to determine what acceptance factors they find most important for a virtual assistant and gain insights into value-adding applications. The unified theory of acceptance and use of technology (UTAUT) was used to structure perceptions and was inductively modified as a result of the interviews. The subsequent research model was triangulated via an online survey with 127 respondents diagnosed with cancer. A structural equation model was used to determine the relevance of acceptance factors. Through a multigroup analysis, differences between sample subgroups were compared.

**Results:**

The interviews found support for all factors of the UTAUT: performance expectancy, effort expectancy, social influence and facilitating conditions. Additionally, self-efficacy, trust, and resistance to change, were added as an extension of the UTAUT. Former patients found a virtual assistant helpful in receiving information about logistic questions, treatment procedures, side effects, or scheduling appointments. The quantitative study found that the constructs performance expectancy (ß = 0.399), effort expectancy (ß = 0.258), social influence (ß = 0.114), and trust (ß = 0.210) significantly influenced behavioral intention to use a virtual assistant, explaining 80% of its variance. Self-efficacy (ß = 0.792) acts as antecedent of effort expectancy. Facilitating conditions and resistance to change were not found to have a significant relationship with user intention.

**Conclusions:**

Performance and effort expectancy are the leading determinants of virtual assistant acceptance. The latter is dependent on a patient’s self-efficacy. Therefore, including patients during the development and introduction of a VA in cancer treatment is important. The high relevance of trust indicates the need for a reliable, secure service that should be promoted as such. Social influence suggests using doctors in endorsing the VA.

**Supplementary Information:**

The online version contains supplementary material available at 10.1186/s12913-022-08189-7.

## Introduction

### Virtual Assistants

The shortage of professionals in the healthcare sector combined with the COVID-19 crisis, increasing digitalization, connectivity, and focus on patient engagement, raise the need for technological improvements in health services [1]. A virtual assistant (VA) is expected to contribute to these goals. VAs have been implemented recently in healthcare settings, though mostly experimental [2–4]. According to Keyser et al. [5] a VA is an embodied or disembodied conversational agent that allows a user to communicate with a technical system in natural language with verbal and/or non-verbal communication characteristics through artificial intelligence (AI) [6, 7]. VA is a synonym for chatbot [5], but for clarity we’ll only use the term VA. VAs are scalable, easily accessible, and operate at low cost and at any time [8, 9]. This makes them a promising tool to increase productivity by managing routine administrative tasks, improving care delivery, and engaging patient in managing their health [10]. Some well-known examples of virtual agents are Google Assistant, Amazon Alexa and Apple’s Siri [11]. In healthcare VAs are used to book appointments, share information and even recommend care based on symptoms [12].

### Current applications of virtual assistants in healthcare

VAs have been analyzed in a variety of functions in healthcare and cancer treatment [13]. Firstly, they assist the treatment success, information, and education of users. Tudor Car et al. [13] enlist an overview of studies reporting on conversational agents to deliver remote services for a wide range of diseases. Among others, they are applied to educate users about sexual health, medication and general health inquiries. For instance, a smartphone VA application to optimize the monitoring of older cancer patients and increase the efficiency of the follow-up process was implemented [14]. The study found the implementation feasible in the target population (geriatric oncology, average age 83 years). Support for cancer patients was delivered through the VA Vik that provides information about breast cancer, its side effects, treatment, and practical information such as patient rights. Two other studies found that patients share surprisingly much with the VA. It improved their adherence rate, provided helpful treatment information and support [15, 16]. Likewise, iDecide, a VA delivering similar information about prostate cancer, significantly increased prostate cancer knowledge and self-efficacy in making informed decisions and using technology [17].

Secondly, VAs are used for self-diagnosis. Most are generic, but also include cancer diagnosis. For instance, two separate studies [18, 19] demonstrate how such a symptom checking VA can be developed, evaluated, and applied. In a later comparative study, the application of the above-mentioned VA [18] displayed a diagnostic accuracy similar to human doctors and safer medical triage advice [20]. Likewise, a VA with a high accuracy rate to diagnose sexually transmitted infections was developed [21], bypassing the need to visit a clinic. A widely used VA in China was used to diagnose conditions involving privacy or stigma issues [22]. Another study [23] found that patients used a symptom checker most commonly to understand the cause of their symptoms and saw it as a helpful diagnostic tool receiving useful information. Generally, a VA offering self-diagnosis requires sensitive data and has profound consequences, leading to higher legal requirements [24]. While the high accessibility, accuracy, and anonymity have led to their increased popularity, many lack the functions to assist the whole diagnostic process of a traditional medical examination [25]. With regards to cancer diseases, self-diagnosis VAs were used for genetic counselling in two studies [26, 27] that report a VA’s viability to assess hereditary cancer risk by querying participants’ family history. The systems showed high engagement, possibly reducing the data collection burden for providers and reaching a broad audience due to geographical and temporal accessibility. This is in line with similar studies proofing the usability of VAs to automate the self-anamnesis, i.e., personal medical history collection, while increasing patient’s motivation to participate [28–31].

Thirdly, VAs improving mental health are highlighted as a separate domain due to their wide use, impact, and relevance for cancer patients, who are susceptible to depression and mood disorders [32]. VAs can improve cancer patients’ wellbeing through mental health assistance and social participation, as studies point to the little time Dutch physicians have for further communication with patients [33, 34]. Up to a third of cancer patients will develop a depression or anxiety disorder and experience problems with daily living, underlining the importance of psychosocial care [35–37]. Nevertheless, these needs are often unmet in secondary care due to an under-identification of psychosocial problems [35]. As a result, patients lack information, which would help them cope and be more involved [38]. Similarly, in a study of Dutch colon cancer patients, respondents were unhappy with psychosocial care. Surgeons agreed that these issues received little attention due to a lack of time and expertise [39]. The COVID-19 pandemic has exacerbated this, as people living with cancer suffer from an increased risk of developing depression during COVID-19 due to feelings of isolation [40]. Moreover, Dutch breast cancer patients reported a substantial drop in emotional and social functioning due to the pandemic [41].

In a review of existing academic research on VAs in a mental health setting [42], a high overall satisfaction and potential for psychiatric use are identified. The effectiveness for patients with major depressive disorders demonstrates a VA’s feasibility with clinical populations. Patients rated the therapeutic alliance between a VA significantly higher than with a clinician [43]. Others disclosed more sensitive information due to anonymity [44]. The self-diagnosis health app Ada was an efficient diagnostic screening or help function for mental disorders in adulthood with the potential to support diagnosticians [45]. For young adults who have undergone cancer treatment, a VA delivering positive psychology skills was perceived as helpful and reduced anxiety [46]. Nevertheless, common standards of reporting and evaluation of VAs are needed for detailed comparisons [42]. Hence, more evidence for the potential of VAs to improve mental health issues and more robust results are needed [47]. A second (scoping) review on VAs in relation to mental health [48] concluded that current results on practicability, feasibility and acceptance of VAs in health care are promising, but especially these topics require more research.

### Adoption of a virtual assistant in health care

**Fig. 1:**
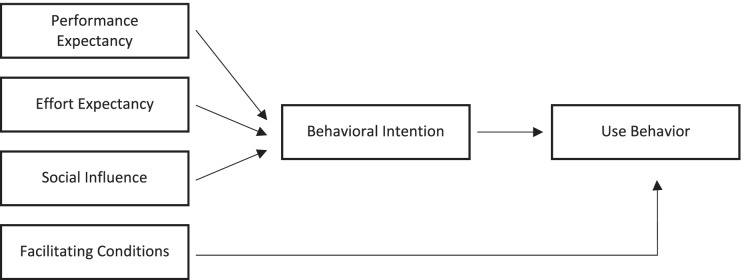
Unified theory of acceptance and use of technology [51]

The adoption of Health Information Systems depends on user perceptions [14, 49]. This implies that for VAs to realize any benefit in health care, patients need to be willing to adopt them and thus have positive perceptions towards them. Here, a holistic conceptual approach is often neglected [50]. To systematize attitudes, several technology acceptance models have been established. Venkatesh et al. [51] compared and integrated eight models resulting in the Unified Theory of Acceptance and Use of Technology (UTAUT). While it is the prevalent framework for healthcare service adoption [22] and has been applied in mobile health [52–54] and telemedicine [50, 55] acceptance research, among others, only indirect research in the context of cancer treatment, an important domain in healthcare, was found [56]. The model consists of four determinants of intention and usage shown in Fig. 1.

First, performance expectancy relates to the beliefs that using a specific technology will help to attain gains, for instance in managing one’s health. Second, effort expectancy means the ease associated with the use of the technology. Third, social influence refers to the degree to which the participant perceives important others, i.e. the social environment, to advocate using a VA. Fourth, facilitating conditions are the degree to which someone believes organizational and technical resources are in place to assist the use of a technology [51].

A range of studies applied the UTAUT to a patient sample and analyzed electronic or mobile health technology acceptance. For instance, research on the adoption of mobile health services among the elderly [53] found that performance expectancy, effort expectancy, and social influence significantly affected behavioral intention, whereas facilitating conditions had no significant relation to intention. The authors explained this could be due to the setting of a developing country, where the elderly were dependent on their children for support. Moreover, they found that two extensions of the model, technology anxiety and resistance to change, could negatively influence intention. This is in line with previous findings that technology anxiety and resistance to change are negatively associated with the technology acceptance model equivalents of effort expectancy and performance expectancy, respectively [57].

Similarly, computer anxiety was found to act as an antecedent with a negative influence of effort expectancy [50]. The authors aimed to predict the determinants of older users’ acceptance of telehealth services with an extended UTAUT model. Performance expectancy, effort expectancy, facilitating conditions and perceived security as addition had a direct influence on intention. Additionally, the doctor’s opinion showed an indirect effect, whereas social influence was no significant predictor [50]. A recent study analyzing the factors influencing telehealth acceptance in Indonesia [58] found similar results. As above, social influence was not significantly associated with behavioral intention, whereas the doctor’s opinion affected performance expectancy and computer anxiety the effort expectancy.

UTAUT has been validated in the context of internet- and mobile-based interventions [59]. The original predictors of acceptance (performance expectancy, effort expectancy and social influence) were found to be true but did not have a moderating effect on acceptance of internet- and mobile-based interventions. Here, internet anxiety was identified as moderator and predictor. Performance expectancy was found to have the strongest influence on acceptance [59].

The notion of perceived security seems especially important considering the present setting, where patients would entrust the VA with sensitive, private information and may ask for medical advice [50]. Therefore, the UTAUT was extended with insights from trust theory to predict the uptake of an AI-based medical diagnosis support system [22]. Besides performance expectancy, initial trust was found the strongest predictor of the behavioral intention of usage. Although the sample in this study consisted of healthcare professionals limiting its transferability, it seems reasonable that trust and security-related concerns are more important in healthcare applications where risks may be more salient than increases in wellbeing. After all, the relationship between safety and innovation seems to be complementary, as discussed in many healthcare fields [60].

While testing the acceptance of telemedicine equipment, all four factors of the UTAUT (performance expectancy, effort expectancy, social influence and facilitating conditions) were found to predict behavioral intention [55]. Yet, clinicians, as well as patients, were included in the sample. Later, an extended model analyzing the acceptance of VAs in healthcare [61] was developed. It includes newly identified constructs such as privacy risk and trust. However, these findings came from interviews with students who mostly had experience in using VAs.

Considering the variety of significant factors, different samples, cultural backgrounds and researched technologies, it is difficult to derive an extended UTAUT that applies accurately to the present research subject. Cancer treatment differs from general healthcare in several points. Firstly, most people newly diagnosed with cancer are 65 years or older [62], implying possibly different priorities for technology adaption [63]. Secondly, besides known side effects, cancer therapies might accelerate the functional decline and lead to psychological distress even after treatment [14, 62]. Thus, the threatening nature of a cancer diagnosis might result in different expectations towards the scope of a digital support tool. Thirdly, though the formation of tumor specific multidisciplinary teams in many regions has improved the coordination of cancer care, alignment of efforts and communication between the various involved providers is far from perfect [14, 64].

The main conclusion from the breadth of results is that the acceptance model must be modified depending on the specific use and target population.

Many studies analyzing a VA’s acceptance in healthcare deliver mixed results and mostly use a digitally literate student sample with a risk of selection bias [65]. Moreover, a large body of research on acceptance in the mobile health context also failed to apply a theoretical base or framework to guide the identification of relevant drivers [66]. In those studies that did use the UTAUT to analyze patient acceptance, factors were of different importance in each case, and other new elements were identified. The variety of diverse and agent-dependent feedback underlines the need for a tailored design according to the targeted population [65]. Additionally, studies lacked further analysis regarding real-life applications and the actual integration [13].

### Virtual Assistants for cancer patients

To summarize, VAs are scalable, easily accessible and operate at low cost at any time [8, 9]. They have been used widely for commercial purposes and have been shown in studies to be beneficial in health care settings. New ways to reach and treat people with cancer are needed. Especially mental health needs during cancer treatment are often neglected and can be hard to fulfil [36, 67]. VAs are a way for health care providers (such as hospitals) to achieve this and acceptance of a VA is a crucial part to achieve adoption [13–47]. Also, the need for further research on the acceptance of VAs in the actual clinical setting has been brought forward [48].

Therefore, our research applies a framework-based analysis to determine acceptance factors in the setting of cancer treatment. This exploratory research examines the acceptance factors and (value-adding) applications of a VA for cancer patients by applying a the UTAUT framework tailored to cancer patients. The research is conducted in cooperation with Maastro, a radiotherapy clinic in the Netherlands, that provides treatment to approximately 250 cancer patients per day.

This leads to the following research questions:

1. What value-adding applications of a VA can a hospital introduce for cancer patients?
2. What are the key factors that drive the acceptance of a VA for cancer patients?

 

## Methods

In this research we applied a mixed-methods approach. The study has a sequential exploratory design. First exploratory qualitative interviews with (cancer) patients and medical professionals, i.e. radiotherapists and physician assistants, from Maastro, were conducted. Possible value-adding applications of a VA were discussed via use cases (research question 1). From the same interviews factors that are most relevant for this target group were identified from the analysis of these interviews. With these factors an extended UTAUT model was formulated. Next, the hypotheses of the model were tested by means of a quantitative survey. The results of the survey were analyzed and lead to the key factors that drive the acceptance of a VA for cancer patients (research question 2).

Prior to the interviews and survey the research protocol was submitted to and approved by Maastro’s Institutional Review Board and the Medical Ethics Committee of Maastricht University Hospital.

### Qualitative in-depth interviews

Participants of in-depth interviews were presented with exemplary interactions with a VA. The contents differed according to the three mentioned areas: (1) information and education, (2) self-diagnosis of symptoms, and (3) mental health. Such demonstrations allowed for more specific feedback and insights into the possible added value for a VA for cancer patients.

#### Data collection

All interviews were conducted online via Microsoft Teams due to the COVID-19 pandemic at the time of the research. Conducting interviews through video calls has been found to be a feasible alternative to in-person qualitative studies [68]. All calls were recorded using Microsoft Teams. The interviews were conducted in German or English depending on the participant’s choice. A native Dutch speaker was present in all meetings for support and translation.

Interviews were setup following the criteria of the qualitative content analysis methodology [69]. This allows building on existing findings while also identifying new patterns [70, 71]. The interviews were guided in a semi-structured way as suggested by previous literature to ensure consistency while also clarifying the complex, novel issue [72, 73]. To identify meta themes in the interviews at least six interviews were needed [74]. The interview guideline was designed so that all participants (former patients and doctors) could first think broadly about attitudes towards a VA and not be influenced by prior applications while later being able to provide specific opinions and recommendations based on an example. Overall, the guideline consists of four segments and builds on elements of similar previous research [61, 75]. The first introductory segment focused on explaining the study background, the functionality of a VA and gathers descriptive information. In the second section, interviewees were asked about potential use cases and (dis)advantages of a VA reflecting on their treatment (patients) or work experience (employees). In the third section, participants were shown a video in which the interaction with a Maastro specific exemplary VA on a smartphone was shown in three different contexts: information and education, self-diagnosis and mental health. In the fourth and last segment, participants were then asked to evaluate the demonstrated VA regarding the helpfulness, design and (dis)advantages. Additionally, they were asked why they would or would not use such a VA and what would convince them to do so.

Former patients also provided insights about value adding applications of a VA by discussing use cases (research question 1). The purpose of demonstrating an exemplary conversation with the Maastro VA was to make the technology more tangible and allow for specific comments. Thus, more credible inputs on use cases and VA acceptability could be gathered [76]. It might have been challenging to elicit actionable findings from qualitative interviews based solely on a description of a new and complex technology that participants primarily did not know. The exemplary conversations were informed by Maastro’s treatment guide [77], the American Cancer Society [78] and the mental health VA Woebot, whose efficacy to reduce depression and anxiety was proven in peer-reviewed literature [79]. The video content was translated to Dutch and lasted four minutes, evenly distributed among the three contexts.

##### Interviews with former patients

Only adult former (cancer) patients of Maastro were eligible to participate in the interviews. A former patient is defined as a patient who had at least one radiotherapy treatment at Maastro, but either does not need further treatment or has been referred. They have completed the patient journey and can therefore offer more holistic insights. They were required to be adults and competent. Interviewees were recruited through self-selection sampling and snowball sampling. These allow contacting the difficult to reach population [80]. First, members of Maastro’s patient council were contacted. The panel represents the interests of patients and keeps track of current developments. Thus, members cannot only share their own experiences but also take other aspects into account. Also, the patient council members were asked to recruit former patients at Maastro from their own network. Third, an invitation to participate in the study was posted on the Maastro Facebook page.

All respondents were informed about the background, structure and duration of the interview via e-mail. It explicitly stated that it is not required to have any experience with VAs, to gather a range of patients. The novelty of the research topic reduces the likelihood that only patients with strong negative or positive prior experience participate. Before the interviews, all former patients signed an extensive declaration of consent.

##### Interviews with radiotherapists and physician assistants

A preselected group of 14 radiotherapists and physician assistants (out of a total of 28) that work at Maastro were invited via e-mail to join the interviews. Preselection was performed pragmatically by their manager based on availability during the scheduled weeks in which the interviews had to be conducted.

#### Data analysis

All interviews were transcribed verbatim. The resulting transcripts were analyzed. were coded based on a deductively formulated category system following the UTAUT factors. Inductively, new information on acceptance was marked by open coding and later grouped by overarching themes [71, 81] to extend the model with additional constructs [69]. A similar approach to tailor the UTAUT has been used previously [61, 82] in different research contexts. Lastly, respondent quotes were selected for the defined themes.

### Quantitative survey

A self-administered online survey design was chosen for this research due to the multitude of identified variables influencing acceptance, the cost-effectiveness of the design and timely data collection [83].

#### Data collection

Potential users of a supportive VA around cancer treatment were targeted for this survey. Only patients that were in the process of being treated, had previously finished their treatment or were awaiting the start of their treatment at Maastro were eligible for participating in the survey.

To recruit them, voluntary and purposive non-probability sampling, namely self-selection sampling, was used. The Facebook account of Maastro shared a post detailing the research background with the weblink to the survey, who the survey was aimed at and a call to participate. Additionally, Maastro patients in active treatment received a printed version of the questionnaire and explanations together with the standard issued weekly form to report side effects (i.e. patient reported outcome measures). Also, the same post with the weblink to the survey was distributed in several Dutch, English, and German thematically appropriate Facebook groups, such as cancer support communities. Because of this approach the size of the potential group of (former) patients that could have responded cannot be determined exactly. It is estimated to be in the order of tens of thousands of people since Maastro treats approximately 7000 patients annually.

The answers were collected over a period of about one month to limit influences resulting from a changing external environment and reduce threats to internal validity [80].

Potential respondents were informed about the duration of the questionnaire and that no names, patient numbers or IP-numbers were asked or logged automatically. An introductory page thanked the respondents for their participation and described the study background, impact, and functionality of a VA to ensure a basic understanding. Two images of an example conversation with a VA like the Maastro specific mock-up served as an illustration enhancing tangibility and comparability. The content related to general information about the treatment modalities and side effects, including the possibility of contacting the doctor.

The survey was created in English. The design of the survey included three to four questions for each acceptance factor. Table 1 shows the survey questions per construct, what their reliability is and from what previous study the questions were derived.

**Table 1:** Survey measurements

Construct	Questions	Source Reliability^a^	Source
Performance expectancy	- I would find a virtual assistant useful during my treatment - Using a virtual assistant would enable me to solve my needs faster - A virtual assistant would improve my treatment experience - If I use a virtual assistant, I will increase my chances of a smooth treatment	ICR: 0.91–0.92^b^	Venkatesh et al. (2003) [51]
Effort expectancy	- My interaction with the virtual assistant would be clear and understandable - Learning how to use a virtual assistant would be easy for me - I would find a virtual assistant easy to use	ICR: 0.90–0.94^b^	Venkatesh et al. (2003) [51]
Social influence	- Peers and other patients would support me using a virtual assistant - My doctor would support me using a virtual assistant - People who are important to me would want me to use a virtual assistant	ICR: 0.88–0.94^b^ (Venkatesh et al., 2003 [51]) CR: 0.93 (Cimperman et al., 2016 [50])	Venkatesh et al. (2003) [51] Adapted by Cimperman et al. (2016) [50] for healthcare
Facilitating Conditions	- I have the resources necessary to use a virtual assistant - I can get help from others if I have difficulties using the virtual assistant - A virtual assistant is compatible with other technologies I use	ICR: 0.83—0.87^b^ (Venkatesh et al., 2003 [51])	Venkatesh et al. (2003) [51]; Venkatesh et al. (2012) [84]
Control question	- The result of this study is impacted by whether participants read instructions carefully. Please indicate that you read the instructions by selecting "agree."		Oppenheimer et al. (2009) [85]
Behavioral intention	- Assuming a virtual assistant is offered, I would intend to use it - I would use a virtual assistant frequently - Given that I had access to a virtual assistant, I would use the services	ICR: 0.93 (Venkatesh et al., 2012) [84] CR = 0.92 (Cimperman et al., 2016) [50]	Venkatesh et al. (2012) [84]; Cimperman et al. (2016) [50]

^a^Internal consistency reliability (ICR), Composite Reliability (CR), Cronbach’s Alpha (CA)

^b^Depending on different time periods

The established factors of the UTAUT were surveyed based on the original work of Venkatesh et al. [51]. For the construct social influence, healthcare-specific modifications [50] were integrated to increase the relevance and fit. Lastly, for the outcome variable behavioral intention, items of Venkatesh et al. [84] and similar adaptions of Cimperman et al. [50] were used. All question formulations were adapted to the application of a VA in healthcare. Each item was measured on a seven-point Likert scale, with answers ranging from “strongly agree” [1] to “strongly disagree” [7]. In addition, an instructional manipulation check was added [85]. In the setting of this study, only behavioral intention can be measured as a dependent variable and not actual usage, because the actual VA has not been developed yet and is described conceptually because it is in a design phase. Relevant studies in the healthcare sector have found facilitating conditions to also directly influence intention [50, 55, 86]. Though deviating from the original model, facilitating conditions is therefore included as a factor in the proposed model.

Gender, age (by decade) and country of residence were collected via the survey. Moreover, respondents were asked whether they previously used an AI-based assistant. Finally, respondents were asked about their treatment status (about to start, completed, currently being treated or neither of the former).

The survey was created in English. A pre-test with five participants led to slight changes in one question. Afterwards, the survey and exemplary VA images were additionally translated to German and Dutch by professional translators.

#### Data analysis

All survey data was checked against the inclusion and exclusion criteria. Incomplete surveys were removed. Also, results from respondents who had completed the survey, but had indicated in the survey they had never received treatment were removed from the data. Finally, those who did not correctly answer the control question (indicating the instructions are read) were excluded.

Next, the results of the survey were analyzed in two stages. In the first stage the measurement model was evaluated. Indicator reliability was assessed by examining the respective item loadings on the constructs and the construct’s internal consistency reliability was evaluated using Jöreskog’s [87] composite reliability, where higher values represent higher levels of reliability and results between 0.70 and 0.95 indicating “satisfactory to good” levels [88]. The model's construct validity, which comprises the convergent and discriminant validity [89], was also assessed.

The Fornell-Larcker criterion was used for evaluation, specifying that the square root of the average variance extracted for each construct should be higher than its correlations with other constructs [90]. This measure was found unsuitable for homogenous loading patterns [91]. As an alternative, the heterotrait-monotrait ratio was suggested [91], which we then applied. In addition, since all the data are collected from a single source, common method bias (CMB) testing was used.

In the second stage the hypotheses were tested through the proposed structural model [92]. Firstly, Harman’s one factor test was applied by performing an unrotated principal factor analysis on all the measurement items in our model [93]. Secondly, the full collinearity assessment [94] was used. For testing the research hypotheses a structural equation model (SEM) was used, more specifically a partial least squares SEM (PLS-SEM). This model poses minimal demands on sample size and measurement scales compared to other SEM techniques. It is a nonparametric, component-based analysis in contrast to popular covariance-based methods [95]. This makes it best suited when analyzing models with formative constructs and small samples [96–98] when using the appropriate thresholds [99]. PLS-SEM was therefore also used in other previously mentioned research exploring technology acceptance [22, 57]. The tool SmartPLS (v. 3.3.3) was used to perform the tests. The measurement quality was evaluated to assess the model’s usability and continue with the structural theory.

As the last step, a multigroup analysis was conducted to evaluate moderation within the model across multiple relationships. Thus, differences between subsamples can be detected with path coefficients directly calculated for each subgroup [100]. Before conducting the multi-group analysis, the measurement invariance of composite models had to be established for each of the three comparisons. It comprised the assessment of (1) configural invariance, (2) compositional invariance and (3) composite equality and results in full, partial or no invariance for constructs [101].

## Results

### Qualitative in-depth interviews

#### Former patients

Eight former patients were interviewed in the first stage: three members of the Maastro patient counsel, who in turn recruited three former patients from their personal network. And finally, two former patients responded to the request on Facebook. Interviewees had a mean age of 58.5 years. The average time between their last treatment and the interview was 5.6 years. Information on the interviewed former patients can be found in Table 2. All interviewees were native Dutch speakers.

**Table 2:** Details of interviewed former patients

Patient	Age	Type of cancer	Year of Treatment
P1	66	Ductal carcinoma in situ (pre-invasive breast cancer)	2013
P2	71	Colorectal cancer	2010
P3	68	Breast cancer	2015 – 2016
P4	37	Breast cancer	2019
P5	64	Laryngeal cancer	2016 – 2017
P6	64	Breast cancer	2015
P7	35	Vaginal cancer	2016
P8	63	Breast Cancer	2017

##### Acceptance factors

*Performance Expectancy:* Statements that are linked to performance expectancy were mentioned as the most important factor to use a VA. The VA was perceived to influence the quality of the treatment in various ways. Two positive aspects are the speed and accessibility of information. One former patient stated, “that you get your information very quickly and you can do it from home” (P4). Likewise, P7 would use a VA, “if the waiting time is long and you would get help with the VA quickly”. In a similar vein, the accessibility of information independent of time and place was seen as a benefit. One can get answers “whenever you like, whenever there are questions” (P3) also “at the weekends, at night” (P5). Consequently, P3 would be convinced to use a VA by his “need for information”, as he “quickly forgot most of the information that they [the doctors] told.” This touches upon the retrievability of information. As P4 put it: “I think it's nice because you have the information, and you can also read it later.” Others “found it difficult to keep all the information in mind” (P3), whereas with a VA “you don’t have to remember all the information” (P2).

In addition, former patients mentioned that anxiety before the treatment could be reduced through more information and better preparation. Further, P5 noted that a VA could lead to improvements by an unbiased assessment, as it “doesn't rely on experience and personal opinion.” Yet, former patients expected answers to be too general or inadequate. P8 summarized the influence of performance expectancy: “If it can help you and your treatment, why not use it then?”.

The first hypothesis based on the UTAUT is, therefore:

1. Performance expectancy has a positive influence on the behavioral intention to use a VA.

*Effort Expectancy:* The ease associated with the use of the technology was mentioned as a critical factor, specifically linked to the imperfect understanding a VA shows during interaction with a human. P7 illustrated, “I live in Limburg, and Google Assistant doesn't understand us because we are supposed to speak like the people in Amsterdam.” Former patients wanted to be easily able to clarify and ask follow-up questions. Thus, for P2, “it's easier to talk to someone […] who knows exactly what you're asking or want to know.” A connection to the input mode was made, as P3 saw the difficulty that “you have to type your answers and it takes some time.” Therefore, she would find it easier to command the VA via voice recognition. As a result, P2 would use the VA “if it is easy to use.” P5 also highlighted the need for “user-friendliness; it should act in the way you expect it at that moment.” Moreover, P5 demanded a “platform independent” user experience. To conclude, the second hypothesis is:

H2: Effort expectancy has a positive influence on the behavioral intention to use a VA.

*Social Influence:* Former patients supported the statement that social influence influenced the degree to which they would use a VA. P7 saw potential in a VA helping her manage medication, “as long as the doctor approves that.” P4 believed the doctor would see a VA critically and would want to maintain regular personal contact besides the VA. The third hypothesis is:

H3: Social influence has a positive influence on the behavioral intention to use a VA.

*Facilitating Conditions:* As facilitating conditions former patients primarily referred to the need for infrastructure to make VAs conveniently accessible from their phone. Specifically, P1 would consider using a VA because “I know that I could use it on my smartphone.” P4 would try a VA as “your phone is something that you always have with you.” As previously outlined, only behavioral intention have been measured as the dependent variable. Therefore, the third hypothesis is:

H4: Facilitating conditions have a positive influence on the behavioral intention to use a VA.

In addition to the established influences, former patients regarded other factors important to the acceptance of a VA in the interviews. As a result, three additional constructs were added to the framework.

The first was self-efficacy, which refers to the perceived ability to acquire expected information and conduct a specific behavior [102]. It does not influence behavioral intention directly but is a predictor of effort expectancy. In the technology acceptance model, self-efficacy is a major determinant of perceived ease of use, conceptually like effort expectancy in the UTAUT [103]. This significant role was also found when investigating the acceptance of mobile health services [102] and information technology by hospital personnel [86].

*Self-efficacy:* Former patients referred to their self-efficacy, including a decreased perceived ability and higher effort due to age or disadvantages. P3 thought a VA is helpful; however, “it will be very strange, […] because my generation isn't used to it.” Similarly, P8 thought that “older people are not so used to work with this.” P3 also mentioned that the ease of using the VA might be lower for people with reading disabilities. Accordingly, self-efficacy concerns have been reported a barrier to mobile health intervention uptake among older adults [104]. Therefore, high self-efficacy was expected to lead to greater ease and freeness in efforts to learn the use of a VA.

H5: Self-efficacy has a positive influence on the effort expectancy of using a VA.

The second construct that resulted from the interviews was trust. It refers to the degree to which a patient is willing to believe in and depend on the VA [22, 105]. The UTAUT lacks this contextual predictor, which is especially important in an inherently relational system like healthcare [106]. For other technologies such as wearable commerce [107], a fitness app [108], electronic commerce [109], as well as mobile banking [110] and payments [111], trust was also found to influence behavioral intention significantly.

*Trust:* P2 stated the precondition that “I have to be able to trust the answers.” Underlying trust expected characteristics are competence, openness and reliability [112, 113]. These were also found in quotes from the interviews. For example, for P1, the primary quality of a VA needs to be “the correctness of the recommendation. […] One must certainly be able to trust him [the VA].” P5 highlighted a similar aspect of competence and wished for openness: “I would like to have a confirmation of what I interacted with in the virtual assistants […] I trust a machine less than a human.” As a result, trust was added as a factor directly influencing behavioral intention:

H6: Trust has a positive influence on the behavioral intention to use a VA.

Lastly, participant’s resistance to change was found to influence the acceptance of a VA. Bhattacherjee and Hikmet [114] defined the acceptance as the preference for the status quo and “generalized opposition to change engendered by the expected adverse consequences of change”. They also argued that the existing acceptance models focused on positive enabling factors but missed negative, inhibiting perceptions. Subsequent research confirmed this relationship [53, 115].

*Resistance to change:* As a VA changes the way patients deal with treatment relevant issues and acquire information, resistance to change came forward during the interviews. P2 interrupted when explaining the study background, stating: “Bad, bad, bad. I'd rather talk to you than a virtual assistant.” Later, when asked about use cases, he could not “think of any” and did “not see any advantage”, whereas “everything is a disadvantage.” Thus, he stated: “a doctor replaced by an assistant; I don't want to imagine that.” P5 would consider a VA to gather information, yet he stated: “if I have a choice, I always take the phone and make a phone call.” Other former patients similarly emphasized wishes to retain the status quo. The seventh hypothesis is:

H7: Resistance to change has a negative influence on the behavioral intention to use a VA.

**Fig. 2:**
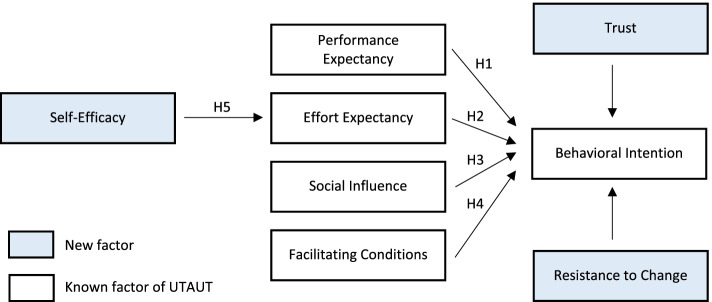
Research model of the extended UTAUT

Figure 2 shows the resulting research model of the extended UTAUT.

##### Use cases 

At the start of the interview, many could imagine the VA helping with administrative tasks such as scheduling appointments (P1, P5, P7) or providing information about the treatment, side effects and medication (P1, P3, P6, P7, P8). This use was seen positively as it was hard to remember the large amount of information in a very emotional situation. In addition, it was mentioned as a means to reduce anxiety before the treatment (P3). P1 could imagine a VA helping with minor issues, as it is like an online search.

After seeing the exemplary mock-up, former patients also liked other conceivable applications, underlining the topic's intangibility. Nonetheless, all of them preferred the two use cases (1) information & education and (2) self-diagnosis of the VA, with the first one being mentioned slightly more often as most helpful (P1, P2, P4, P5, P7). A common theme here was that they liked “factual information” (P2) and would use it to get information about the treatment (P3, P5, P8). While the first context relating to general information was thus perceived very well, the second with more sensitive content about side effects as part of a self-diagnosis was controversial. While some perceived it as dangerous or scary (P5) and found it “less good” (P2), others saw this one as “most helpful” (P3).

The third application relating to mental health was seen most critically, with P1, P2, P3, P4 and P8 ranking it least helpful due to a lack of empathy and compassion. As P3 put it: “I would find it difficult to speak about mental health, about emotions, with a virtual assistant. For this, the human component is important.”

#### Radiotherapists and physician assistants

##### Acceptance factors

Four radiotherapists out of 14 radiotherapists and physician assistants at Maastro accepted and were interviewed subsequently to extend these insights with an operational perspective. Their mean age is 43.5 years. Their specialization and age are presented in Table 3 below. Each interview lasted approximately 30 min.

**Table 3:** Details of interviewed radiotherapists and physician assistants

Doctor	Age	Field of specialization
D1	55	Breast cancer
D2	34	Neuropsychological tumors
D3	39	Prostate cancer
D4	46	Gastrointestinal tumors, breast cancer

Interviews with doctors of the radiotherapy institute confirmed the findings from the interviews with former patients as they touched upon similar aspects. Again, performance expectancy was found particularly salient as VAs could adequately answer standard questions and might be more convenient due to their accessibility. D2 was convinced that “most patients are eager to do what benefits their health. And if we would present this to them, most patients would just accept it […].” Regarding effort expectancy, doctors added that the language of a VA must be understandable, as healthcare professionals’ communication is often too complex for patients to comprehend. Again, trust in the capabilities was imperative for them to use and recommend usage to patients. Concerns regarding the self-efficacy and resistance to change of some patients were further mentioned.

##### Use cases

The doctor’s attitudes towards the conversations illustrated in the video were like those of patients. Information delivery about the clinic, location and treatment was seen as most helpful with the best chance to be implemented (D1, D2, D4). D1 recapitulated: “So, the first example: where do I have to be and what's going to happen, I think that's perfect. That can be very easily done and is widely applicable in healthcare.” The assessment of symptoms as part of self-diagnosis was seen more critically, as perceived risks and interventions were more decisive in this area. While D2 thought it could be most helpful, D3 found “it can be dangerous”. In this light, he emphasized the need to “build in enough red lights” and positively mentioned the referral to the doctor. Lastly, the mental health context was seen as potential support (D1, D3). However, again, there were concerns about the feasibility due to a lack of empathy and compassion (D1, D2, D4).

In general, doctors saw the technology as an addition, complementary to current initiatives, which could support patients in specific areas and thus free up time (D1, D3).

Firstly, they frequently mentioned the delivery of information not directly related to the treatment. D4 thought a VA “can take away a lot of the more logistical questions: How can I travel to the clinic? How often do I have to come?” Similarly, D2 imagined that it could help with questions about the process “like taxi, appointments”.

Secondly, doctors envisioned a VA to prepare patients for their treatment. D3 stated it could help give patients a clear idea of what to expect by illustrating the treatment environment (D3). This is consistent with comments from patients who stated that a VA could reduce anxiety before treatment. Further, D4 explained that most preparatory information is “quite standard”, so “more or less the same for every patient”, which is why this can be automated. D2 also stated that walking patients through their upcoming treatment would be helpful. A VA could potentially do this better “because patients just have more time to think about the options that they have.”

Thirdly, a VA was mentioned to add value for patients during the treatment by digitizing the side effect assessment and recording. D4 thought a VA could be “an easier or a nicer way” to fill out the patient-reported outcome measurement list, which collects information about experienced side effects due to the higher interaction than the current pen and paper method. Further, D1 and D2 agreed that most questions regarding side effects are similar so that a VA could answer standard ones. Therefore, D3 thought a VA could “easily support or replace one consultation [out of four] […] once people are under treatment” (D3). Lastly, D3 also thought of a use for doctors whereby a VA prioritizes patients based on the stated severity of the side effects to select when to see which patient.

### Quantitative survey

The initial list of survey measurements was extended with questions that were based on the three additional constructs derived from the interviews. Table 4 provides an overview of the added questions per construct, the study they were derived from and its reliability.

**Table 4:** Extended survey measurements

Construct	Questions	Source Reliability^a^	Source
Trust	- I would trust a virtual assistant to be reliable - I believe a virtual assistant could provide secure services I believe a virtual assistant is trustworthy	CA: 0.969	Chandra et al. (2010) [116]
Resistance to change	- I don’t want virtual assistants to change the way I deal with treatment-related problems - I don’t want virtual assistants to change the way I get treatment-related information - I don’t want a virtual assistant to change the way I interact with hospital employees - Overall, I don’t want virtual assistants to change my treatment	CR: 0.92	Bhattacherjee & Hikmet (2007) [114]
Self-efficacy	- It is convenient for me to use a virtual assistant - A virtual assistant would be convenient to use for me - I am able to use the virtual assistant without much effort	CR: 0.892	Zhang et al. (2017) [102]

^a^Composite Reliability (CR), Cronbach’s Alpha (CA)

The construct trust was measured by adapting the scale of Chandra et al. [116] from the research context of mobile payments adoption. Items for the resistance to change were adopted from a study that analyzed physician’s resistance to healthcare technology [114]. Self-efficacy was assessed using items from Zhang et al. [102], who adapted the theory of Johnston and Warkentin [117] to evaluate the acceptance of mobile health services.

#### Respondents

After applying the exclusion criteria as described in the methodology section, the dataset contained 127 valid responses. Characteristics of the survey respondents are shown in Table 5. Respondents resided in the Netherlands [63], Germany [32], the USA [25] and United Kingdom [7]. The gender was approximately evenly distributed, with 59 male and 68 female respondents. The average age of the respondents was 61.4 years. Therefore, a sample consisting of mostly elderly was used in addition to the precondition of a cancer diagnosis, in contrast to some previous acceptance studies using digitally literate student samples. Respondents’ experience with a VA differed: 49 of them never used a VA before, while 42 some form of prior experience and 36 were unsure if they had ever used a VA.

**Table 5:** Characteristics of survey respondents

Country of Residence	Netherlands	Germany	USA	UK		
	63 [50%]	32 [25%]	25 [20%]	7 [6%]		
Gender	Male	Female				
	59 [46%]	68 [54%]				
Age	< 30	31–40	41–50	51–60	61–70	> 70
	2 [2%]	6 [5%]	20 [16%]	22 [17%]	49 [39%]	28 [22%]
Experience	Yes	No	Unsure			
	42 [33%]	49 [39%]	36 [28%]			

#### First stage: analysis of the measurement model

Table 6 shows the reliability and convergent validity for the measurement model.

**Table 6:** Reliability and convergent validity

Construct	Item	Loading	Composite reliability	Cronbach’s Alpha	Average variance extracted
Behavioral Intention	BI1	0.953	0.959	0.936	0.887
	BI2	0.923			
	BI3	0.949			
Effort Expectancy	EE1	0.865	0.928	0.883	0.811
	EE2	0.912			
	EE3	0.925			
Facilitating Conditions	FC1	0.912	0.911	0.853	0.773
	FC2	0.826			
	FC3	0.899			
Performance Expectancy	PE1	0.929	0.958	0.942	0.851
	PE2	0.934			
	PE3	0.927			
	PE4	0.901			
Resistance to Change	RtC1	0.907	0.948	0.927	0.820
	RtC2	0.932			
	RtC3	0.930			
	RtC4	0.851			
Self-Efficacy	SE1	0.835	0.926	0.879	0.807
	SE2	0.938			
	SE3	0.919			
Social Influence	SI1	0.795	0.871	0.787	0.693
	SI2	0.827			
	SI3	0.873			
Trust	TR1	0.911	0.953	0.927	0.872
	TR2	0.935			
	TR3	0.955			

Regarding indicator reliability all outer loadings were above the suggested threshold of 0.7, ranging from 0.795 to 0.955 [98, 99]. The construct’s internal consistency reliability was found to range from 0.871 to 0.959. While high reliability and correspondingly values close to 1 are generally desirable, results above 0.95 can be problematic as they might indicate semantical redundancy in the items [118]. However, this was ruled out for the present work, as the formulations were closely taken from existing academic literature with only slight adaptations. Also, in a related study [50], composite reliability scores of 0.97 were accepted. For the model's construct validity, constructs' average variance extracted ranges from 0.693 to 0.887 and is above the threshold of 0.50, indicating that each construct explains more than half of the items’ variance [98, 119]. Discriminant validity found the square root of the average variance extracted for each construct to be higher than its correlations with other constructs, as presented in Table 7.

**Table 7:** Fornell-Larcker criterion for PLS-SEM

	Behavioral Intention	Effort Expectancy	Facilitating Conditions	Performance Expectancy	Resistance to Change	Self-Efficacy	Social Influence	Trust
**Behavioral Intention**	**0.942**							
**Effort Expectancy**	0.795	**0.901**						
**Facilitating Conditions**	0.586	0.731	**0.879**					
**Performance Expectancy**	0.843	0.782	0.486	**0.923**				
**Resistance to Change**	-0.307	-0.219	-0.214	-0.236	**0.906**			
**Self-Efficacy**	0.750	0.792	0.798	0.613	-0.215	**0.899**		
**Social Influence**	0.447	0.347	0.169	0.456	0.161	0.260	**0.832**	
**Trust**	0.735	0.607	0.544	0.684	-0.322	0.597	0.327	**0.934**

For the heterotrait-monotrait ratio no confidence interval with the upper bound at 95% for the constructs contained the value one, thus confirming discriminant validity [108]. The heterotrait-monotrait intervals are shown in Table 8.

**Table 8:** Heterotrait-monotrait confidence intervals

	**Original Sample**	**Sample Mean**	**5.0%**	**95.0%**
Effort Expectancy—> Behavioral Intention	0.876	0.876	0.811	0.931
Facilitating Conditions—> Behavioral Intention	0.655	0.655	0.506	0.791
Facilitating Conditions—> Effort Expectancy	0.830	0.829	0.746	0.901
Performance Expectancy—> Behavioral Intention	0.897	0.896	0.842	0.942
Performance Expectancy—> Effort Expectancy	0.861	0.861	0.797	0.918
Performance Expectancy—> Facilitating Conditions	0.536	0.537	0.361	0.698
Resistance to Change—> Behavioral Intention	0.328	0.331	0.142	0.501
Resistance to Change—> Effort Expectancy	0.242	0.251	0.129	0.388
Resistance to Change—> Facilitating Conditions	0.234	0.244	0.120	0.386
Resistance to Change—> Performance Expectancy	0.255	0.262	0.086	0.427
Self-Efficacy—> Behavioral Intention	0.829	0.829	0.734	0.908
Self-Efficacy—> Effort Expectancy	0.897	0.897	0.826	0.957
Self-Efficacy—> Facilitating Conditions	0.915	0.915	0.823	0.990
Self-Efficacy—> Performance Expectancy	0.676	0.676	0.548	0.785
Self-Efficacy—> Resistance to Change	0.238	0.242	0.110	0.374
Social Influence—> Behavioral Intention	0.494	0.492	0.349	0.625
Social Influence—> Effort Expectancy	0.387	0.395	0.233	0.566
Social Influence—> Facilitating Conditions	0.219	0.252	0.122	0.424
Social Influence—> Performance Expectancy	0.494	0.493	0.338	0.639
Social Influence—> Resistance to Change	0.197	0.220	0.098	0.384
Social Influence—> Self-Efficacy	0.286	0.310	0.202	0.437
Trust—> Behavioral Intention	0.784	0.785	0.665	0.879
Trust—> Effort Expectancy	0.671	0.673	0.553	0.788
Trust—> Facilitating Conditions	0.617	0.618	0.488	0.734
Trust—> Performance Expectancy	0.728	0.731	0.590	0.848
Trust—> Resistance to Change	0.344	0.345	0.208	0.469
Trust—> Self-Efficacy	0.659	0.661	0.534	0.775
Trust—> Social Influence	0.356	0.360	0.203	0.511

Finally, CMB was tested. Harman’s one factor test found more than one factor and the largest factor did not account for a majority of the variance (only 27.2%). Collinearity assessment showed that all the variance inflation factors from the full collinearity assessment did not exceed the suggested threshold of 3.33 [120]. Based on the results of the two assessments we conclude that CMB is not an issue in our study.

Concludingly, the reliability and validity of the measurement model were established.

#### Second stage: analysis of the structural model

To test the formulated hypotheses, the PLS-SEM evaluated the predictive capabilities of the structural model. The PLS-SEM *can* be a path model when there are only manifest variables, but it also allows for latent variables (that are inferred by the manifest variables). SEM extends the traditional path analysis with confirmatory factor analysis to test whether specific data fits a hypothesized measurement model (i.e. construct validity) [96, 121]. Hence, PLS-SEM fits with the intended research purpose and the analysis shows the following results.

Firstly, all variance inflation factor values for the research model were below the threshold five, thus ruling out multicollinearity [98]. The model further showed an acceptable fit with the saturated model's standardized root mean square residual slightly below the conservative threshold of 0.8 [91]. The saturated model in which all constructs can freely correlate was used following the recommendation of Benitez et al. [122].

The dependent variable behavioral intention was explained with an R^2^ of 0.806. The second dependent variable, effort expectancy showed an R^2^ of 0.628, i.e., nearly 63% of its variance is explained by self-efficacy. While the R^2^ measures accuracy in terms of in-sample prediction, Q^2^ was, to some extent, a measure of out-of-sample prediction [98, 123]. Here, an omission distance of seven, as recommended by Hair et al. [124], yielded a Q^2^ value for behavioral intention of 0.696 and 0.499 for effort expectancy. These results confirm the high predictive accuracy and relevance of the model [96].

The significance and strength of the path coefficients, shown in Table 9, were assessed to test the proposed hypotheses. Of the initial seven hypotheses, five were accepted. As expected, performance expectancy (ß = 0.399, *P* < 0.001) and effort expectancy (ß = 0.258, *P* = 0.003) had a significant, strong and positive influence on behavioral intention. social influence (ß = 0.114, *P* = 0.046) further showed predictive significance for behavioral intention. Contrary, facilitating conditions revealed no significant influence on behavioral intention. The newly introduced variable trust (ß = 0.210, *P* = 0.006) also significantly influenced behavioral intention, whereas resistance to change (ß = -0.097, *P* = 0.059) was only marginally significant. Lastly, self-efficacy (ß = 0.792, *P* < 0.001) significantly predicted effort expectancy. In addition, Table 9 displays the effect sizes f^2^ of the variables. It is a measure of magnitude independent of sample size with values less than 0.02 indicating no relevant effect, as is the case for facilitating conditions [122]. Even though resistance change was only marginally significant, there might have still been a small effect [98].

**Table 9:** Results of the structural components model

Hypothesis		Path coefficient	T Statistic	P Value	Effect size f^2^	Decision
H1	Performance Expectancy --> Behavioral Intention	0.399	3.814	** < 0.001**	0.220	Supported
H2	Effort Expectancy --> Behavioral Intention	0.258	2.965	**0.003**	0.077	Supported
H3	Social Influence --> Behavioral Intention	0.114	1.996	**0.046**	0.047	Supported
H4	Facilitating Conditions --> Behavioral Intention	0.050	0.718	0.473	0.005	Not supported
H5	Self-Efficacy --> Effort Expectancy	0.792	19.878	** < 0.001**	1.686	Supported
H6	Trust --> Behavioral Intention	0.210	2.761	**0.006**	0.102	Supported
H7	Resistance to Change --> Behavioral Intention	-0.097	1.888	0.059	0.039	Not supported

Results from a SEM were based on an analysis of a single population and failed to evaluate differences between subgroups [125]. A multi-group analysis in a PLS-SEM was applied to test for moderating variables and return group-specific path coefficients, thereby minimizing the potential for misrepresentation [100]. In this article, two groups of age, gender, and experience were compared, as shown in Table 10.

**Table 10:** Subgroups for the multigroup analysis

Age		Gender		Experience	
Group 1: < 61 years	Group 2: ≥61 years	Group 1: male	Group 2: female	Group 1: prior use	Group 2: no prior use
*n* = 55	*n* = 77	*n* = 59	*n* = 68	*n* = 42	*n* = 49

A multi-group analysis was conducted for constructs that show at least partial invariance (Table 11) [126]

**Table 11:**
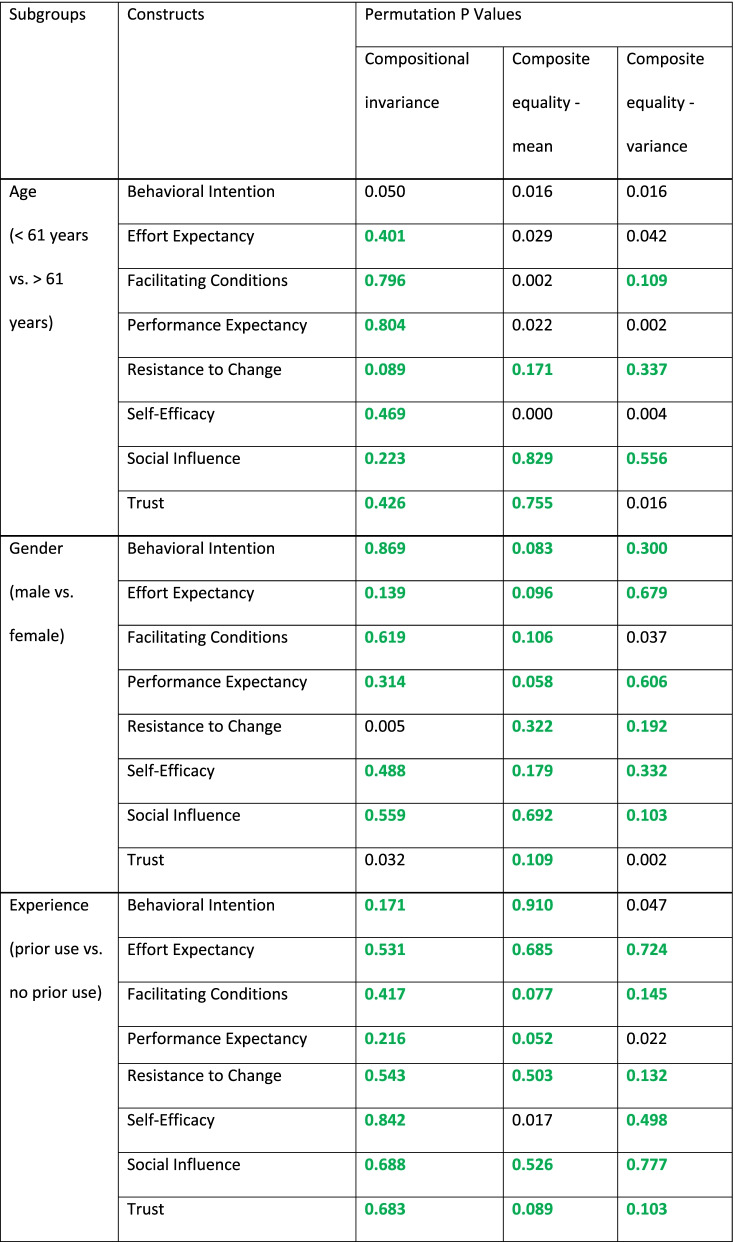
Results of steps two and three of the measurement invariance for the composite models test

**Fig. 3:**
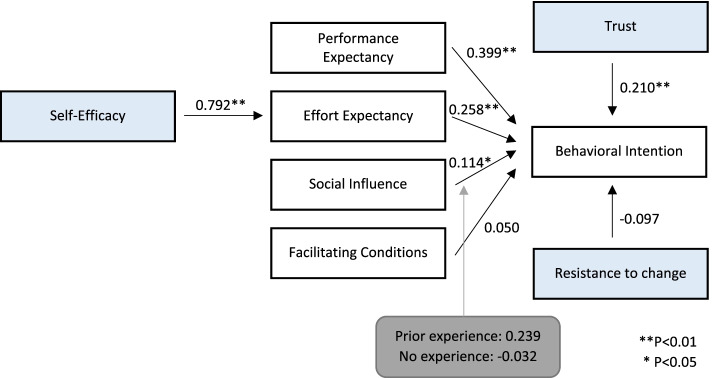
Structural model and path coefficients

The results of the three multi-group analyses comparing age, gender and experience subgroups were primarily insignificant, with only two marginally significant differences regarding the impact of prior experience on the path from social influence on intention, as well as the impact of facilitating conditions on intention across the two gender groups (Table 12). The results are as displayed in Fig. 3.

**Table 12:** Multigroup analysis results

		**Path Coefficients Group 1**	**Path Coefficients Group 2**	**Path Coefficients Difference**	**P Value**
Age (< 61 years vs. > 61 years)	Effort Expectancy—> Behavioral Intention	0.346	0.199	0.147	.455
	Facilitating Conditions—> Behavioral Intention	-0.116	0.090	-0.206	.181
	Performance Expectancy—> Behavioral Intention	0.218	0.468	-0.250	.242
	Resistance to Change—> Behavioral Intention	-0.086	-0.096	0.010	.953
	Self-Efficacy—> Effort Expectancy	0.742	0.805	-0.064	.442
	Social Influence—> Behavioral Intention	0.144	0.109	0.035	.788
	Trust—> Behavioral Intention	0.369	0.176	0.193	.334
Gender (male vs. female)	Effort Expectancy—> Behavioral Intention	0.202	0.334	-0.132	.498
	Facilitating Conditions—> Behavioral Intention	0.175	-0.089	0.264	.058
	Performance Expectancy—> Behavioral Intention	0.272	0.401	-0.129	.525
	Resistance to Change—> Behavioral Intention	-0.146	-0.089	-0.057	.678
	Self-Efficacy—> Effort Expectancy	0.753	0.825	-0.073	.362
	Social Influence—> Behavioral Intention	0.081	0.152	-0.071	.521
	Trust—> Behavioral Intention	0.285	0.205	0.080	.567
Experience (prior use vs. no prior use)	Effort Expectancy—> Behavioral Intention	0.159	0.131	-0.190	.354
	Facilitating Conditions—> Behavioral Intention	0.037	0.042	-0.063	.680
	Performance Expectancy—> Behavioral Intention	0.577	0.564	0.340	.144
	Resistance to Change—> Behavioral Intention	-0.209	-0.195	-0.070	.537
	Self-Efficacy—> Effort Expectancy	0.753	0.760	0.072	.486
	Social Influence—> Behavioral Intention	0.276	0.240	0.308	.052
	Trust—> Behavioral Intention	0.069	0.100	-0.279	.162

## Discussion

### Main findings

Former cancer patients and radiotherapists stated various value-adding applications that a hospital can introduce for a VA. Patients considered providing treatment-related information such as treatment-logistics, common side effects and the treatment procedure itself the most promising application of a VA. They expressed mixed feelings about the use of a VA for diagnosis with some patients considering this helpful, while others regarded this a dangerous application. Radiotherapists expected VAs to reduce their workload by deploying a VA to answer common questions from patients about side effects and the treatment procedure. Furthermore, they saw the potential of a VA for acquiring (digitalized) patient reported outcome measures. Both radiotherapists and former patients expected this to offer more or faster reassurance for patients, thus reducing anxiety.

The interviews pointed to performance expectancy and effort expectancy as the key factors that drive acceptance of a VA. Former patients positively highlighted the speed, accessibility and objectivity when providing information. A user-friendly design was stated as an important factor for effort expectancy. This factor is also influenced by the self-efficacy [102] of the user. Two extensions of the research model were Trust was found an important facilitator and participant’s resistance to change as an inhibitor to accepting a VA. We extended the UTAUT model with these factors.

To quantify and validate this extended model, a survey was completed by 127 cancer patients. The strongest factors that drive the VA’s acceptance among cancer patients are performance expectancy (ß = 0.399), effort expectancy (ß = 0.258) and trust (ß = 0.210). Social influence (ß = 0.114) showed a positive though weaker influence on behavioral intention.

Overall, descriptive statistics indicated a willingness to adopt a VA for cancer treatment. The average aggregated value for behavioral intention was 3.2 measured on a 7-point scale from “strongly agree” [1] to “strongly disagree” [7]. This was more pronounced among younger respondents. A multi-group analysis did not find significant differences in the relevance of constructs among the subgroups age, gender and experience with a VA. Reasons for the insignificant differences between the subgroups might have been the partly unequal sample distribution and small sample sizes leading to insufficient power to detect differences [127].

### The extended UTAUT model: hypotheses and perspectives

Compared to Venkatesh et al.’s [51] study, the present model confirmed the three predictors of behavioral intention in H1, H2 and H3. As the performance expectancy construct is the strongest predictor in each of the different acceptance models [51] and specifically for internet- and mobile-based interventions [59] its high value and support for H1 was expected in our study. The weaker effect of effort expectancy compared to performance expectancy fits previous research [128] that found ease of use the secondary motivation to adopt a technology. The primary motivator is the function of the tool itself. In other words: ease of use cannot compensate for unnecessity. The lower relevance of social influence could be because older users are less receptive to societal pressure or image [129]. Furthermore, the UTAUT was initially introduced in an organizational environment where peer influence is more salient [130].

Unexpectedly, facilitating conditions showed no significant influence on behavioral intention and H4 is not supported. In the original UTAUT, facilitating conditions is considered having direct effect on user behavior instead of intention [51]. This insignificance might be due to the unawareness of the elderly about the technical and infrastructural resource requirements of a VA [53]. In addition to the existing model, the antecedent self-efficacy showed a highly significant, positive relation with effort expectancy, explaining 63% of its variance. H5 is thus supported. Therefore, the high perceived capabilities of the user were also crucial for the acceptance of a VA through an indirect effect, confirming previous research [102].

The high relevance of the factor trust underlined the sensitive issue of a VA during cancer treatment, which bears extensive consequences on people’s lives. Accordingly, trust played a central role for healthcare professionals in acceptance research [22, 131]. Also, in the present study, H6 was supported. As trust involves some degree of risk [132], patients only relied on a VA when their level of confidence about the reliability (TR1), security (TR2) and trustworthiness (TR3) is higher than the perceived risks involved. Lastly, resistance to change only had a marginally significant influence. H7 is therefore not supported. This result is unexpected, as the inhibitor was observed in the interviews and particularly salient in studies with older participants [53, 115]. A reason for the marginal significance can be related to the dominate and overwhelming effects of performance expectancy, efficacy expectancy and trust in the overall model. Another reason could be attributed to our data collection, which was partly through Facebook. This might have introduced a bias for respondents that are more open to new technological innovations. Moreover, patients may already proactively met information needs, e.g., through mobile online searches, so a VA would not have represented a major intervention.

### Theoretical contributions

This study contributes to technology acceptance theory by analyzing the behavioral intention to use a VA from a the perspective of cancer patients – a perspective that is often overlooked [133, 134]. This study validates the UTAUT model in cancer patients based on empirical qualitative and quantitative fieldwork insightsand extends it with two new antecedents of effort expectancy. *Trust was* found to be a highly factor of influence (highlighting its relevance in healthcare services), while *resistance to change* was not found to play an important role. Also, while the UTAUT model [51] included age, gender and experience as moderating variables, it was not tested in previous studies applying the framework, e.g., [22, 50, 53, 86]. Lastly, this article addresses the call for more conceptual approaches to acceptance research [50, 59] by analyzing attitudes towards VAs as a new service for cancer patients though such a service does not exist yet. This study attempts to forecast acceptance by quantifying perceptions rather than assessing reasons for failed initiatives.

### Managerial Implications

To be accepted, a VA must be useful and provide value in demanded areas where there are frictions in the patient journey. Thus, insights from patient-oriented research should guide the design. As we found performance expectancy the strongest determinant for the acceptance of a VA, this should be the primary target when trying to increase acceptance among cancer patients. Also, performance expectancy has been found to benefit treatment for mental disorders as a primary mechanism that affects change [135]. This may be assumed to be true for cancer patients (with mental-health related issues) and therefore a relatively direct way to improve their well-being. Contrary to these findings, in our study former patients expressed their concerns about using a VA with mental health issues as they expected the ‘human component’ and empathy to be missing. Further research on this topic is adviced. Interventions to increase acceptance, e.g. educational [136] or informational [137], have been shown effective, but should be tailored to the specific target population, i.e. cancer patients.

Older people might prefer voice input [138] when using a VA, especially in the case of visual impairment [139]. However, the focus should not only lay on the attributes of a VA but also those of the users. Our study found patients will find a VA easy to use only when they have the perceived self-efficacy, leading to a need for training and close involvement in introducing a VA. In UTAUT, the normative social influence tends to encourage compliance or acceptance [140]. The social influence factor may lead to diffusion of VA usage from early, mostly younger patients to other, less affine ones. In a clinical setting, patients feel the importance that if others, in particular doctors, believe the patient should use a specific technology [141]. This suggests using doctors to advocate the uptake of a VA. It is particularly salient as older people rely on their opinion and often avoid or refer decision-making to compensate for a reduced risk tolerance [142]. Trust can be increased by incorporating feedback from patients and the patient’s council in the development and by including options such as a referral to the doctor in the interaction with a VA. An open, standard evaluation framework, as currently developed by the World Health Organization, would help build trust and select value-adding applications [143].

### Limitations and further research

The above empirical results need to be evaluated against the backdrop of some limitations. Only a few former patients of the interviews received their treatment up to 11 years ago, limiting the transferability of their experience and integration of a VA into it. Moreover, although members of the patient council can provide a holistic view, they may be satisfied above average with the treatment as they are still voluntarily working with the clinic. Therefore, they might see fewer points for improvement retrospectively. Unfortunately, it was not possible to recruit more former patients of Maastro within the timeframe of the study, given the ethical and privacy restrictions that are related to clinical research. Likewise, there is a risk of selection bias as it was not possible to sample former patients representatively regarding cancer types and age groups. Instead, self-selection and snowball sampling are used. This similarly applies to doctors, limiting generalizability. Further, the content and online means of showing an illustrative example might have influenced interviewees' opinions. Although, similar interview questions about a VA were asked after only a general description of the concept. Nonetheless, further research should replicate this study with a realistic, interactive VA. Due to language barriers, some former patients may have been discouraged to participate or reluctant to elaborate. To decrease these effects, a native Dutch speaker was present in the interviews.

The sampling procedure of the quantitative research limits its external validity. The survey was mainly distributed via Facebook. Thus, only people with an account and a certain degree of digital affinity could partake, excluding particularly some elderly. Moreover, including respondents residing in a multitude of countries limits explanatory power. Yet, it was necessary to achieve a large enough sample. Here, future research may use only one nationality. Furthermore, insignificant differences between subgroups could be due to the small sample size. Future research with the ability to recruit larger samples should test whether the findings hold across subgroups.

The self-selection in online surveys carries the risk that primarily those who have intrinsic motivation and interest participate. Other facets might have been lost due to this from people more averse to these innovations, e.g., regarding the factor resistance to change which was only marginally significant. Handing out a printed version to all patients with the side effect sheet may reduce self-selection to a certain extent. Additionally, the research was cross-sectional conducted in a short period. A longitudinal study accompanying the introduction will provide added value by measuring the actual use behavior omitted in this study and the potential change of constructs such as performance expectancy and effort expectancy over time.Moreover, trust formation can be split and analyzed at different levels [144]. Therefore, further studies should assess the antecedents of trust. For instance, it may test whether a VA promoted as “approved by doctors” or “developed by a provider with years of experience” creates more trust.

## Conclusion

While the capabilities of VAs are well recognized in other industries, this study provides novel insights into the use and perception of a VA for cancer patients. The findings can assist healthcare providers in identifying value-adding applications and increasing acceptance of a VA. A VA can offer benefits, especially for mental well-being, by changing how the treatment is managed and how information is delivered to patients. VAs are also expected to reduce the workload for health care providers by answering certain patient questions. Building on an extended UTAUT model that was derived from interviews with former cancer patients and radiotherapists, key factors that drive the acceptance of a VA for cancer patients were determined: performance expectancy, effort expectancy, and trust must be addressed for a VA to be successfully adopted by cancer patients. Patients only find a VA easy to use when they have the perceived self-efficacy, which requires training. Also, doctors that actively advocate a VA are expected to increase the uptake. And finally, including patients in the development of a VA helps to increase trust. Academically, this study extends the technology acceptance research in healthcare in general and for cancer patients specifically.

## Supplementary Information

**Additional file 1.** Interview Guideline.

**Additional file 2.** An exemplary mockup of conversations with the Exemplary Virtual assistant.

## Data Availability

The datasets used and/or analyzed during the current study are available from the corresponding author on reasonable request. Data is not publicly available due to the privacy of participants.

## Abbreviations

AI: Artificial intelligence
PLS: Partial least squares
SEM: Structural equation model
UTAUT: Unified theory of acceptance and use of technology
VA: Virtual assistant
