# Supplementary material for: Analyzing the determinants to accept a virtual assistant and use cases among cancer patients: a mixed methods study
Source: BMC Health Serv Res. 2022 Jul 9;22:890. doi: 10.1186/s12913-022-08189-7 (PMC9270807; doi:10.1186/s12913-022-08189-7)
Supplement: Supplementary file 1 — Additional file 1. Interview Guideline. [file 12913_2022_8189_MOESM1_ESM.docx]

### Additional file 1: Interview Guideline

| **Section** | **Question** |
| --- | --- |
| Section 1: Introduction & experience | - *Thanking participants for their willingness to help and to improve the situation for future patients.* - *Informing participants about the procedure, informed consent, their rights* - *Definition of a* virtual assistant (*VA) and illustration of how it operates* - *Gathering demographic information (age, type of cancer)* |
|  | - Did you use a VA before? (e.g., Alexa, Google Home, etc.) - In which context did you use them? - Describe your experience with the VA? |
| Section 2: Broad, open questions on VA in healthcare | - What application scenarios of a VA can you imagine at Maastro considering your treatment? - In which area do you think virtual assistants will substitute doctors one day? Why not? - What do you think could be the advantages and disadvantages of using a VA during the treatment? - What problems could a VA solve, and which problems could occur as a result of using them? |
| Section 3: Demonstration video | *Showing video of an interaction with an exemplary Maastro VA relating to three different contexts: (1) information & education, (2) self-diagnosis, (3) mental health* |
| Section 4: Key questions and evaluation | - Which of the three application areas did you find most helpful? Why? - After seeing the Maastro VA, would you use such an assistant? If yes, why? If not, why not? - What factors of how a chatbot is designed and applied are essential from your point of view? - Considering the use of the Maastro VA, what advantages and disadvantages do you see? - What would convince you to use the Maastro VA? |
