# Supplementary figures and images for: Analyzing the determinants to accept a virtual assistant and use cases among cancer patients: a mixed methods study
Source: BMC Health Serv Res. 2022 Jul 9;22:890. doi: 10.1186/s12913-022-08189-7 (PMC9270807; doi:10.1186/s12913-022-08189-7)

###
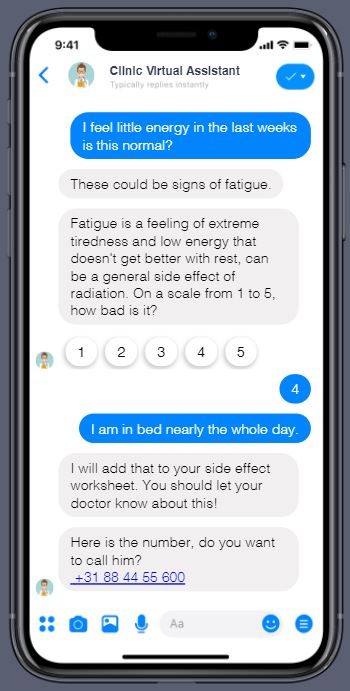
Additional file 2: Conversations with the Exemplary Virtual assistant


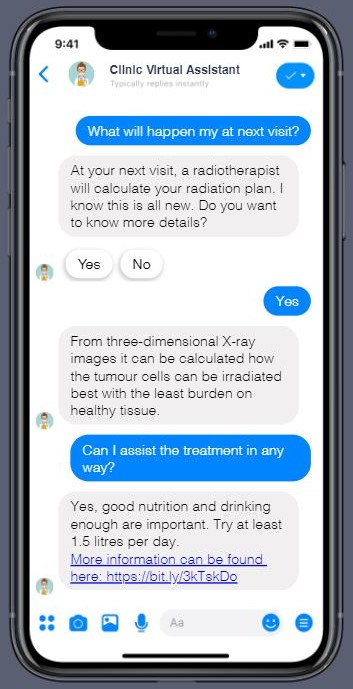

Supplement: Supplementary file 2 — Additional file 2. An exemplary mockup of conversations with the Exemplary Virtual assistant. [file 12913_2022_8189_MOESM2_ESM.docx]
